# Supplementary material for: End Sequence Analysis Toolkit (ESAT) expands the extractable information from single-cell RNA-seq data
Source: Genome Res. 2016 Oct;26(10):1397–410. doi: 10.1101/gr.207902.116 (PMC5052061; doi:10.1101/gr.207902.116)
Supplement: Supplemental Material [file supp_gr.207902.116_Supp_Materials.tar.gz › ESAT-esat_v0.1/doc/html/classumms_1_1core_1_1model_1_1_alignment_model.html]

ESAT: umms.core.model.AlignmentModel Class Reference


ESAT


- Main Page
- Related Pages
- Packages
- Classes
- Files

- Class List
- Class Index
- Class Hierarchy
- Class Members

All Classes Namespaces Files Functions Variables Enumerator Pages

- umms
- core
- model
- AlignmentModel

Classes |
Public Member Functions |
Protected Attributes |
List of all members

umms.core.model.AlignmentModel Class Reference

Inheritance diagram for umms.core.model.AlignmentModel:

[legend]

Collaboration diagram for umms.core.model.AlignmentModel:

[legend]

|  |  |
| --- | --- |
| Classes | |
| class | AlignmentCount |
|  | |
| class | FilteredIterator |
|  | |
| class | NodeIterator |
|  | |
| class | WrapAlignmentCountIterator |
|  | |

|  |  |
| --- | --- |
| Public Member Functions | |
|  | AlignmentModel (String bamFile, CoordinateSpace coordinateSpace, Collection< Predicate< Alignment >> readFilters, boolean readOrCreatePairedEndBam) |
|  | |
|  | AlignmentModel () |
|  | |
|  | AlignmentModel (String bamFile, CoordinateSpace coordinateSpace, Collection< Predicate< Alignment >> readFilters, boolean readOrCreatePairedEndBam, TranscriptionRead transcriptionRead) |
|  | |
|  | AlignmentModel (String bamFile, CoordinateSpace coordinateSpace, Collection< Predicate< Alignment >> readFilters, boolean readOrCreatePairedEndBam, TranscriptionRead transcriptionRead, boolean fragment) |
|  | |
|  | AlignmentModel (String bamFile, CoordinateSpace coordinateSpace, Collection< Predicate< Alignment >> readFilters, boolean readOrCreatePairedEndBam, TranscriptionRead transcriptionRead, String maskedRegionFile) |
|  | |
|  | AlignmentModel (String bamFile, CoordinateSpace coordinateSpace, Collection< Predicate< Alignment >> readFilters, boolean readOrCreatePairedEndBam, TranscriptionRead transcriptionRead, boolean fragment, String maskedRegionFile) |
|  | |
|  | AlignmentModel (String bamFile, CoordinateSpace coordinateSpace, Collection< Predicate< Alignment >> readFilters) |
|  | |
|  | AlignmentModel (String bamFile, CoordinateSpace coordinateSpace, Collection< Predicate< Alignment >> readFilters, TranscriptionRead transcriptionRead) |
|  | |
|  | AlignmentModel (String bamFile, CoordinateSpace coordinateSpace, boolean readOrCreatePairedEndBam) |
|  | |
|  | AlignmentModel (String bamFile, CoordinateSpace coordinateSpace, boolean readOrCreatePairedEndBam, TranscriptionRead transcriptionRead) |
|  | |
|  | AlignmentModel (String bamFile, CoordinateSpace coordinateSpace) |
|  | |
|  | AlignmentModel (String bamFile, CoordinateSpace coordinateSpace, TranscriptionRead transcriptionRead) |
|  | |
| void | computeGlobalStats () |
|  | |
| CloseableIterator< Alignment > | getReadIterator () |
|  | |
| CloseableIterator< Alignment > | getReadIterator (Annotation region) |
|  | |
| List< Double > | getPositionCountList (Gene gene) throws IOException |
|  | |
| double | getRefSequenceCounts (String refName) |
|  | |
| double | getCount (Annotation window, boolean fullyContained) |
|  | |
| double | getCount () |
|  | |
| double | getCount (CloseableIterator< AlignmentCount > iter) |
|  | |
| double | getGlobalLambda () |
|  | |
| double | getGlobalLength () |
|  | |
| double | getGlobalPairedFragments () |
|  | |
| EmpiricalDistribution | getReadSizeDistribution (CoordinateSpace coord, int maxSize, int numBins) |
|  | |
| EmpiricalDistribution | getReadSizeDistribution (Annotation region, CoordinateSpace coord, int maxSize, int numBins) |
|  | |
| double | getMedianReadSize (Annotation region, CoordinateSpace coord, int maxSize, int numBins, boolean fullyContained) |
|  | |
| double | getMedianReadSize (Annotation region, CoordinateSpace coord, int maxSize, int numBins) |
|  | |
| EmpiricalDistribution | getReadSizeDistribution (Annotation region, CoordinateSpace coord, int maxSize, int numBins, boolean fullyContained) |
|  | |
| CloseableIterator< AlignmentCount > | getOverlappingReadCounts (Annotation region, boolean fullyContained) |
|  | |
| CloseableIterator< Alignment > | getOverlappingSplicedReads (Annotation region, boolean fullyContained) |
|  | |
| CloseableIterator< Alignment > | getOverlappingSplicedReads (String chr) |
|  | |
| CloseableIterator< Alignment > | getOverlappingReads (String chr) |
|  | |
| int | getBasesCovered (Annotation region, boolean fullyContained) |
|  | |
| CloseableIterator< Alignment > | getPermutedAnnotations (Annotation region) |
|  | |
| double | getGlobalNumReads () |
|  | |
| double | getGlobalNumReadsReferenceSeqs () |
|  | |
| boolean | isValid (Alignment read) |
|  | |
| double | getCount (Annotation window) |
|  | |
| boolean | containsReference (String refName) |
|  | |
| double | getGlobalCount () |
|  | |
| double | getGlobalCountReferenceSeqs () |
|  | |
| int | size () |
|  | |
| void | addFilter (Predicate< Alignment > filter) |
|  | |
| void | addFilters (Collection< Predicate< Alignment >> filters) |
|  | |
| WindowScoreIterator< CountScore > | scan (Annotation region, int windowSize, int overlap) |
|  | |
| long | getRefSequenceLength (String refName) |
|  | |
| Iterator<?extends Window > | windowIterator (int windowSize, int overlap) |
|  | |
| double | getRefSequenceLambda (String refName) |
|  | |
| CoordinateSpace | getCoordinateSpace () |
|  | |
| String | getBamFile () |
|  | |
| int | getBasesCovered (Annotation region) |
|  | |
| void | removeFilter (Predicate< Alignment > readFilter) |
|  | |
| int | getIntronCounts (Annotation intron) |
|  | |
| Map< Alignment, Integer > | getOverlappingReadReplicateCounts (Annotation region, boolean fullyContained) |
|  | |
| CloseableIterator< Alignment > | getOverlappingReads (Annotation region, boolean fullyContained) |
|  | |
| Annotation | getPeak (Annotation parent, Annotation region, boolean fullyContained) |
|  | |
| CloseableIterator< Alignment > | getOverlappingAnnotations (Annotation region, boolean fullyContained) |
|  | |
| CloseableIterator< Alignment > | iterator () |
|  | |
| double | getCountExcludingRegion (Annotation region, Annotation excluded) |
|  | |
| double | getSplicedCount (Alignments alignments, boolean fullyContained) |
|  | |
| double | getAverageCountPerPositionWithThreshold (Gene gene, int minCount) |
|  | |
| double | getAverageCountPerPosition (Gene gene) |
|  | |
| double[] | getCountsPerPosition (Gene gene) |
|  | |
| List< Double > | getCountsStrandedPerPosition (Annotation gene) |
|  | |
| double | getCountStrandedExcludingRegion (Annotation region, Annotation excluded) |
|  | |
| Map< String, Integer > | getRefSequenceLengths () |
|  | |
| TranscriptionRead | getStrand () |
|  | |

|  |  |
| --- | --- |
| Protected Attributes | |
| CoordinateSpace | coordinateSpace |
|  | |

## Detailed Description

Serves the purpose of the Generic DataAlignment model and the ContinuousDataAlignmentModel Will populate alignments If a paired end file has unmapped mates, it will be represented by a single end read If both mates are mapped it will be represented as paired end reads Therefore a single model might have both, use flag X to exclude or include only one type This will be a bridge to the analysis modules

Author
:   skadri

## Constructor & Destructor Documentation

|  |  |  |  |
| --- | --- | --- | --- |
| umms.core.model.AlignmentModel.AlignmentModel | ( | String | *bamFile*, |
|  |  | CoordinateSpace | *coordinateSpace*, |
|  |  | Collection< Predicate< Alignment >> | *readFilters*, |
|  |  | boolean | *readOrCreatePairedEndBam* |
|  | ) |  |  |

Build with a BAM file Populate the alignment collection

Parameters
:   |  |  |
    | --- | --- |
    | bamFile |  |
    | coordinateSpace |  |
    | readFilters |  |

Exceptions
:   |  |  |
    | --- | --- |
    | IOException |  |

|  |  |  |  |  |
| --- | --- | --- | --- | --- |
| umms.core.model.AlignmentModel.AlignmentModel | ( |  | ) |  |

|  |  |  |  |
| --- | --- | --- | --- |
| umms.core.model.AlignmentModel.AlignmentModel | ( | String | *bamFile*, |
|  |  | CoordinateSpace | *coordinateSpace*, |
|  |  | Collection< Predicate< Alignment >> | *readFilters*, |
|  |  | boolean | *readOrCreatePairedEndBam*, |
|  |  | TranscriptionRead | *transcriptionRead* |
|  | ) |  |  |

Populate the alignment collection

Parameters
:   |  |  |
    | --- | --- |
    | bamFile |  |
    | coordinateSpace |  |
    | readFilters |  |

Exceptions
:   |  |  |
    | --- | --- |
    | IOException |  |

|  |  |  |  |
| --- | --- | --- | --- |
| umms.core.model.AlignmentModel.AlignmentModel | ( | String | *bamFile*, |
|  |  | CoordinateSpace | *coordinateSpace*, |
|  |  | Collection< Predicate< Alignment >> | *readFilters*, |
|  |  | boolean | *readOrCreatePairedEndBam*, |
|  |  | TranscriptionRead | *transcriptionRead*, |
|  |  | boolean | *fragment* |
|  | ) |  |  |

Populate the alignment collection

Parameters
:   |  |  |
    | --- | --- |
    | bamFile |  |
    | coordinateSpace |  |
    | readFilters |  |

Exceptions
:   |  |  |
    | --- | --- |
    | IOException |  |

|  |  |  |  |
| --- | --- | --- | --- |
| umms.core.model.AlignmentModel.AlignmentModel | ( | String | *bamFile*, |
|  |  | CoordinateSpace | *coordinateSpace*, |
|  |  | Collection< Predicate< Alignment >> | *readFilters*, |
|  |  | boolean | *readOrCreatePairedEndBam*, |
|  |  | TranscriptionRead | *transcriptionRead*, |
|  |  | String | *maskedRegionFile* |
|  | ) |  |  |

|  |  |  |  |
| --- | --- | --- | --- |
| umms.core.model.AlignmentModel.AlignmentModel | ( | String | *bamFile*, |
|  |  | CoordinateSpace | *coordinateSpace*, |
|  |  | Collection< Predicate< Alignment >> | *readFilters*, |
|  |  | boolean | *readOrCreatePairedEndBam*, |
|  |  | TranscriptionRead | *transcriptionRead*, |
|  |  | boolean | *fragment*, |
|  |  | String | *maskedRegionFile* |
|  | ) |  |  |

Populate the alignment collection

Parameters
:   |  |  |
    | --- | --- |
    | bamFile |  |
    | coordinateSpace |  |
    | readFilters |  |
    | tmpDir | Temp directory for sorting |

Exceptions
:   |  |  |
    | --- | --- |
    | IOException |  |

reader cache - AD

Here is the call graph for this function:

|  |  |  |  |
| --- | --- | --- | --- |
| umms.core.model.AlignmentModel.AlignmentModel | ( | String | *bamFile*, |
|  |  | CoordinateSpace | *coordinateSpace*, |
|  |  | Collection< Predicate< Alignment >> | *readFilters* |
|  | ) |  |  |

|  |  |  |  |
| --- | --- | --- | --- |
| umms.core.model.AlignmentModel.AlignmentModel | ( | String | *bamFile*, |
|  |  | CoordinateSpace | *coordinateSpace*, |
|  |  | Collection< Predicate< Alignment >> | *readFilters*, |
|  |  | TranscriptionRead | *transcriptionRead* |
|  | ) |  |  |

|  |  |  |  |
| --- | --- | --- | --- |
| umms.core.model.AlignmentModel.AlignmentModel | ( | String | *bamFile*, |
|  |  | CoordinateSpace | *coordinateSpace*, |
|  |  | boolean | *readOrCreatePairedEndBam* |
|  | ) |  |  |

|  |  |  |  |
| --- | --- | --- | --- |
| umms.core.model.AlignmentModel.AlignmentModel | ( | String | *bamFile*, |
|  |  | CoordinateSpace | *coordinateSpace*, |
|  |  | boolean | *readOrCreatePairedEndBam*, |
|  |  | TranscriptionRead | *transcriptionRead* |
|  | ) |  |  |

|  |  |  |  |
| --- | --- | --- | --- |
| umms.core.model.AlignmentModel.AlignmentModel | ( | String | *bamFile*, |
|  |  | CoordinateSpace | *coordinateSpace* |
|  | ) |  |  |

|  |  |  |  |
| --- | --- | --- | --- |
| umms.core.model.AlignmentModel.AlignmentModel | ( | String | *bamFile*, |
|  |  | CoordinateSpace | *coordinateSpace*, |
|  |  | TranscriptionRead | *transcriptionRead* |
|  | ) |  |  |

## Member Function Documentation

|  |  |  |  |  |  |
| --- | --- | --- | --- | --- | --- |
| void umms.core.model.AlignmentModel.addFilter | ( | Predicate< Alignment > | *filter* | ) |  |

Here is the caller graph for this function:

|  |  |  |  |  |  |
| --- | --- | --- | --- | --- | --- |
| void umms.core.model.AlignmentModel.addFilters | ( | Collection< Predicate< Alignment >> | *filters* | ) |  |

Here is the call graph for this function:

|  |  |  |  |  |
| --- | --- | --- | --- | --- |
| void umms.core.model.AlignmentModel.computeGlobalStats | ( |  | ) |  |

Compute global stats and cache to a file for future use, or read from file if already cached

Here is the call graph for this function:

Here is the caller graph for this function:

|  |  |  |  |  |  |
| --- | --- | --- | --- | --- | --- |
| boolean umms.core.model.AlignmentModel.containsReference | ( | String | *refName* | ) |  |

Here is the caller graph for this function:

|  |  |  |  |  |  |
| --- | --- | --- | --- | --- | --- |
| double umms.core.model.AlignmentModel.getAverageCountPerPosition | ( | Gene | *gene* | ) |  |

Get the average position level coverage

Parameters
:   |  |  |
    | --- | --- |
    | gene | The gene |

Returns
:   The average of all position counts in the gene

Here is the call graph for this function:

|  |  |  |  |
| --- | --- | --- | --- |
| double umms.core.model.AlignmentModel.getAverageCountPerPositionWithThreshold | ( | Gene | *gene*, |
|  |  | int | *minCount* |
|  | ) |  |  |

Get average position level coverage of only positions with count above a threshold (denominator excludes positions not passing threshold)

Parameters
:   |  |  |
    | --- | --- |
    | gene | The gene |
    | minCount | Min coverage to include a position |

Returns
:   The average of all position counts over threshold in the gene

Here is the call graph for this function:

|  |  |  |  |  |
| --- | --- | --- | --- | --- |
| String umms.core.model.AlignmentModel.getBamFile | ( |  | ) |  |

|  |  |  |  |
| --- | --- | --- | --- |
| int umms.core.model.AlignmentModel.getBasesCovered | ( | Annotation | *region*, |
|  |  | boolean | *fullyContained* |
|  | ) |  |  |

Here is the caller graph for this function:

|  |  |  |  |  |  |
| --- | --- | --- | --- | --- | --- |
| int umms.core.model.AlignmentModel.getBasesCovered | ( | Annotation | *region* | ) |  |

Here is the call graph for this function:

|  |  |  |  |  |
| --- | --- | --- | --- | --- |
| CoordinateSpace umms.core.model.AlignmentModel.getCoordinateSpace | ( |  | ) |  |

Here is the caller graph for this function:

|  |  |  |  |
| --- | --- | --- | --- |
| double umms.core.model.AlignmentModel.getCount | ( | Annotation | *window*, |
|  |  | boolean | *fullyContained* |
|  | ) |  |  |

Use the coordinate space to decide what is an overlapping read

Parameters
:   |  |  |
    | --- | --- |
    | window |  |
    | fullyContained |  |

Returns

Here is the call graph for this function:

Here is the caller graph for this function:

|  |  |  |  |  |
| --- | --- | --- | --- | --- |
| double umms.core.model.AlignmentModel.getCount | ( |  | ) |  |

Use the coordinate space to decide what is an overlapping read Gets the count over the entire genome

Returns

Here is the caller graph for this function:

|  |  |  |  |  |  |
| --- | --- | --- | --- | --- | --- |
| double umms.core.model.AlignmentModel.getCount | ( | CloseableIterator< AlignmentCount > | *iter* | ) |  |

|  |  |  |  |  |  |
| --- | --- | --- | --- | --- | --- |
| double umms.core.model.AlignmentModel.getCount | ( | Annotation | *window* | ) |  |

Here is the call graph for this function:

|  |  |  |  |
| --- | --- | --- | --- |
| double umms.core.model.AlignmentModel.getCountExcludingRegion | ( | Annotation | *region*, |
|  |  | Annotation | *excluded* |
|  | ) |  |  |

Here is the call graph for this function:

|  |  |  |  |  |  |
| --- | --- | --- | --- | --- | --- |
| double [] umms.core.model.AlignmentModel.getCountsPerPosition | ( | Gene | *gene* | ) |  |

This function returns an array list of counts for each position along the specified gene

Parameters
:   |  |  |
    | --- | --- |
    | gene |  |

Returns

Here is the call graph for this function:

Here is the caller graph for this function:

|  |  |  |  |  |  |
| --- | --- | --- | --- | --- | --- |
| List<Double> umms.core.model.AlignmentModel.getCountsStrandedPerPosition | ( | Annotation | *gene* | ) |  |

This function returns an array list of counts for each position along the specified gene

Parameters
:   |  |  |
    | --- | --- |
    | gene |  |

Returns

Here is the call graph for this function:

|  |  |  |  |
| --- | --- | --- | --- |
| double umms.core.model.AlignmentModel.getCountStrandedExcludingRegion | ( | Annotation | *region*, |
|  |  | Annotation | *excluded* |
|  | ) |  |  |

Here is the call graph for this function:

|  |  |  |  |  |
| --- | --- | --- | --- | --- |
| double umms.core.model.AlignmentModel.getGlobalCount | ( |  | ) |  |

Here is the call graph for this function:

Here is the caller graph for this function:

|  |  |  |  |  |
| --- | --- | --- | --- | --- |
| double umms.core.model.AlignmentModel.getGlobalCountReferenceSeqs | ( |  | ) |  |

Here is the call graph for this function:

|  |  |  |  |  |
| --- | --- | --- | --- | --- |
| double umms.core.model.AlignmentModel.getGlobalLambda | ( |  | ) |  |

Here is the call graph for this function:

Here is the caller graph for this function:

|  |  |  |  |  |
| --- | --- | --- | --- | --- |
| double umms.core.model.AlignmentModel.getGlobalLength | ( |  | ) |  |

Here is the call graph for this function:

Here is the caller graph for this function:

|  |  |  |  |  |
| --- | --- | --- | --- | --- |
| double umms.core.model.AlignmentModel.getGlobalNumReads | ( |  | ) |  |

Get total number of reads

Returns
:   Total read count

Here is the call graph for this function:

|  |  |  |  |  |
| --- | --- | --- | --- | --- |
| double umms.core.model.AlignmentModel.getGlobalNumReadsReferenceSeqs | ( |  | ) |  |

Get total number of reads mapping to the coordinate space

Returns
:   Total read count on coordinate space

Here is the call graph for this function:

|  |  |  |  |  |
| --- | --- | --- | --- | --- |
| double umms.core.model.AlignmentModel.getGlobalPairedFragments | ( |  | ) |  |

Here is the call graph for this function:

|  |  |  |  |  |  |
| --- | --- | --- | --- | --- | --- |
| int umms.core.model.AlignmentModel.getIntronCounts | ( | Annotation | *intron* | ) |  |

This will count the number of spliced reads with exactly this intron

Parameters
:   |  |  |
    | --- | --- |
    | intron | the intron to count |

Returns

Here is the call graph for this function:

|  |  |  |  |
| --- | --- | --- | --- |
| double umms.core.model.AlignmentModel.getMedianReadSize | ( | Annotation | *region*, |
|  |  | CoordinateSpace | *coord*, |
|  |  | int | *maxSize*, |
|  |  | int | *numBins*, |
|  |  | boolean | *fullyContained* |
|  | ) |  |  |

Get the median of all read (or fragment) sizes in an annotation

Parameters
:   |  |  |
    | --- | --- |
    | region | The annotation in which to count read sizes |
    | coord | Coordinate space in which to compute read sizes |
    | maxSize | The maximum read size to check for |
    | numBins | The number of bins for histogram |
    | fullyContained | Count fully contained reads/fragments only |

Returns
:   Distribution of read sizes with respect to coordinate space

Here is the call graph for this function:

|  |  |  |  |
| --- | --- | --- | --- |
| double umms.core.model.AlignmentModel.getMedianReadSize | ( | Annotation | *region*, |
|  |  | CoordinateSpace | *coord*, |
|  |  | int | *maxSize*, |
|  |  | int | *numBins* |
|  | ) |  |  |

Get the median of all read (or fragment) sizes in an annotation

Parameters
:   |  |  |
    | --- | --- |
    | region | The annotation in which to count read sizes |
    | coord | Coordinate space in which to compute read sizes |
    | maxSize | The maximum read size to check for |
    | numBins | The number of bins for histogram |

Returns
:   Distribution of read sizes with respect to coordinate space

Here is the call graph for this function:

|  |  |  |  |
| --- | --- | --- | --- |
| CloseableIterator<Alignment> umms.core.model.AlignmentModel.getOverlappingAnnotations | ( | Annotation | *region*, |
|  |  | boolean | *fullyContained* |
|  | ) |  |  |

|  |  |  |  |
| --- | --- | --- | --- |
| CloseableIterator<AlignmentCount> umms.core.model.AlignmentModel.getOverlappingReadCounts | ( | Annotation | *region*, |
|  |  | boolean | *fullyContained* |
|  | ) |  |  |

Return the reads that overlap with this region in coordinate space If the Alignment model has a strand which is NOT unstranded, then returns stranded counts

Here is the caller graph for this function:

|  |  |  |  |
| --- | --- | --- | --- |
| Map<Alignment, Integer> umms.core.model.AlignmentModel.getOverlappingReadReplicateCounts | ( | Annotation | *region*, |
|  |  | boolean | *fullyContained* |
|  | ) |  |  |

Get number of replicates of each read overlapping the region

Parameters
:   |  |  |
    | --- | --- |
    | region | The region |
    | fullyContained | Count fully contained reads only |

Returns
:   For each unique read position, the number of replicates

Here is the call graph for this function:

|  |  |  |  |  |  |
| --- | --- | --- | --- | --- | --- |
| CloseableIterator<Alignment> umms.core.model.AlignmentModel.getOverlappingReads | ( | String | *chr* | ) |  |

Here is the call graph for this function:

Here is the caller graph for this function:

|  |  |  |  |
| --- | --- | --- | --- |
| CloseableIterator<Alignment> umms.core.model.AlignmentModel.getOverlappingReads | ( | Annotation | *region*, |
|  |  | boolean | *fullyContained* |
|  | ) |  |  |

Here is the call graph for this function:

|  |  |  |  |
| --- | --- | --- | --- |
| CloseableIterator<Alignment> umms.core.model.AlignmentModel.getOverlappingSplicedReads | ( | Annotation | *region*, |
|  |  | boolean | *fullyContained* |
|  | ) |  |  |

Return the reads that overlap with this region in coordinate space

Here is the caller graph for this function:

|  |  |  |  |  |  |
| --- | --- | --- | --- | --- | --- |
| CloseableIterator<Alignment> umms.core.model.AlignmentModel.getOverlappingSplicedReads | ( | String | *chr* | ) |  |

Return the reads that overlap with this region in coordinate space

Here is the call graph for this function:

|  |  |  |  |
| --- | --- | --- | --- |
| Annotation umms.core.model.AlignmentModel.getPeak | ( | Annotation | *parent*, |
|  |  | Annotation | *region*, |
|  |  | boolean | *fullyContained* |
|  | ) |  |  |

Get the span covered by all reads overlapping a region

Parameters
:   |  |  |
    | --- | --- |
    | parent | Parent annotation of the region of interest |
    | region | The region of interest |
    | fullyContained | Fully contained reads only |

Returns
:   The subregion of the parent gene enclosing all reads overlapping the region or null if no overlappers

Here is the call graph for this function:

|  |  |  |  |  |  |
| --- | --- | --- | --- | --- | --- |
| CloseableIterator<Alignment> umms.core.model.AlignmentModel.getPermutedAnnotations | ( | Annotation | *region* | ) |  |

Here is the call graph for this function:

|  |  |  |  |  |  |
| --- | --- | --- | --- | --- | --- |
| List<Double> umms.core.model.AlignmentModel.getPositionCountList | ( | Gene | *gene* | ) | throws IOException |

Get list of position level counts in mature transcript

Parameters
:   |  |  |
    | --- | --- |
    | gene | The region |

Returns
:   List of position counts

Exceptions
:   |  |  |
    | --- | --- |
    | IOException |  |

Here is the call graph for this function:

|  |  |  |  |  |
| --- | --- | --- | --- | --- |
| CloseableIterator<Alignment> umms.core.model.AlignmentModel.getReadIterator | ( |  | ) |  |

Here is the caller graph for this function:

|  |  |  |  |  |  |
| --- | --- | --- | --- | --- | --- |
| CloseableIterator<Alignment> umms.core.model.AlignmentModel.getReadIterator | ( | Annotation | *region* | ) |  |

|  |  |  |  |
| --- | --- | --- | --- |
| EmpiricalDistribution umms.core.model.AlignmentModel.getReadSizeDistribution | ( | CoordinateSpace | *coord*, |
|  |  | int | *maxSize*, |
|  |  | int | *numBins* |
|  | ) |  |  |

Get the distribution of all read (or fragment) sizes with respect to a coordinate space

Parameters
:   |  |  |
    | --- | --- |
    | coord | The coordinate space |
    | maxSize | The maximum read size to consider |
    | numBins | The number of bins for histogram |

Returns
:   Distribution of read sizes with respect to coordinate space

Here is the call graph for this function:

Here is the caller graph for this function:

|  |  |  |  |
| --- | --- | --- | --- |
| EmpiricalDistribution umms.core.model.AlignmentModel.getReadSizeDistribution | ( | Annotation | *region*, |
|  |  | CoordinateSpace | *coord*, |
|  |  | int | *maxSize*, |
|  |  | int | *numBins* |
|  | ) |  |  |

Get the distribution of read (or fragment) sizes in an annotation

Parameters
:   |  |  |
    | --- | --- |
    | region | The annotation in which to count read sizes |
    | coord | Coordinate space in which to compute read sizes |
    | maxSize | The maximum read size to check for |
    | numBins | The number of bins for histogram |

Returns
:   Distribution of read sizes with respect to coordinate space

Here is the call graph for this function:

|  |  |  |  |
| --- | --- | --- | --- |
| EmpiricalDistribution umms.core.model.AlignmentModel.getReadSizeDistribution | ( | Annotation | *region*, |
|  |  | CoordinateSpace | *coord*, |
|  |  | int | *maxSize*, |
|  |  | int | *numBins*, |
|  |  | boolean | *fullyContained* |
|  | ) |  |  |

Get the distribution of read (or fragment) sizes in an annotation

Parameters
:   |  |  |
    | --- | --- |
    | region | The annotation in which to count read sizes |
    | coord | Coordinate space in which to compute read sizes |
    | maxSize | The maximum read size to check for |
    | numBins | The number of bins for histogram |
    | fullyContained | Count fully contained reads/fragments only |

Returns
:   Distribution of read sizes with respect to coordinate space

Here is the call graph for this function:

|  |  |  |  |  |  |
| --- | --- | --- | --- | --- | --- |
| double umms.core.model.AlignmentModel.getRefSequenceCounts | ( | String | *refName* | ) |  |

This function calculates the number of proper paired end reads over the entire coordinate space

Author
:   skadri

Returns
:   Returns the counts for the specified reference sequence

Parameters
:   |  |  |
    | --- | --- |
    | refName |  |

Returns

Here is the call graph for this function:

Here is the caller graph for this function:

|  |  |  |  |  |  |
| --- | --- | --- | --- | --- | --- |
| double umms.core.model.AlignmentModel.getRefSequenceLambda | ( | String | *refName* | ) |  |

Here is the call graph for this function:

|  |  |  |  |  |  |
| --- | --- | --- | --- | --- | --- |
| long umms.core.model.AlignmentModel.getRefSequenceLength | ( | String | *refName* | ) |  |

Here is the caller graph for this function:

|  |  |  |  |  |
| --- | --- | --- | --- | --- |
| Map<String, Integer> umms.core.model.AlignmentModel.getRefSequenceLengths | ( |  | ) |  |

Get the lengths of the reference sequences

Returns
:   Map associating each reference name with sequence length

|  |  |  |  |
| --- | --- | --- | --- |
| double umms.core.model.AlignmentModel.getSplicedCount | ( | Alignments | *alignments*, |
|  |  | boolean | *fullyContained* |
|  | ) |  |  |

Here is the call graph for this function:

|  |  |  |  |  |
| --- | --- | --- | --- | --- |
| TranscriptionRead umms.core.model.AlignmentModel.getStrand | ( |  | ) |  |

|  |  |  |  |  |  |
| --- | --- | --- | --- | --- | --- |
| boolean umms.core.model.AlignmentModel.isValid | ( | Alignment | *read* | ) |  |

|  |  |  |  |  |
| --- | --- | --- | --- | --- |
| CloseableIterator<Alignment> umms.core.model.AlignmentModel.iterator | ( |  | ) |  |

|  |  |  |  |  |  |
| --- | --- | --- | --- | --- | --- |
| void umms.core.model.AlignmentModel.removeFilter | ( | Predicate< Alignment > | *readFilter* | ) |  |

Removes a read filter from the set

Parameters
:   |  |  |
    | --- | --- |
    | readFilter | to remove |

|  |  |  |  |
| --- | --- | --- | --- |
| WindowScoreIterator<CountScore> umms.core.model.AlignmentModel.scan | ( | Annotation | *region*, |
|  |  | int | *windowSize*, |
|  |  | int | *overlap* |
|  | ) |  |  |

|  |  |  |  |  |
| --- | --- | --- | --- | --- |
| int umms.core.model.AlignmentModel.size | ( |  | ) |  |

Here is the call graph for this function:

|  |  |  |  |
| --- | --- | --- | --- |
| Iterator<? extends Window> umms.core.model.AlignmentModel.windowIterator | ( | int | *windowSize*, |
|  |  | int | *overlap* |
|  | ) |  |  |

Iterate through the whole coordinate space in windows and score them using the provided window processor

Parameters
:   |  |  |
    | --- | --- |
    | windowSize | size of the windows to score |

## Member Data Documentation

CoordinateSpace umms.core.model.AlignmentModel.coordinateSpace

protected

---

The documentation for this class was generated from the following file:

- java/umms/core/model/AlignmentModel.java


---

Generated on Fri Aug 15 2014 15:11:11 for ESAT by  

 1.8.7
